# Comorbidities and the management of hypertensive heart disease in a low-resource setting: a cross-sectional study

**DOI:** 10.1097/MS9.0000000000002931

**Published:** 2025-03-27

**Authors:** Raïssa K. Kongue, Cody M. Dinganga, Dominique Vervoort, Idriss Mwanaut, Marc Tshilanda, Yvan Zolo, Jonathan A. Nuamah, Princess Y. Benson, Ulrick S. Kanmounye

**Affiliations:** a Department of Internal Medicine, Faculty of Medicine, Bel Campus University of Technology, Kinshasa, Democratic Republic of Congo; b Cardiology Unit, Department of Internal Medicine, Monkole Mother and Child Hospital Center, Kinshasa, Democratic Republic of Congo; c Bloomberg School of Public Health, Johns Hopkins University, Baltimore, Maryland, USA; d Carey Business School, Johns Hopkins University, Baltimore, Maryland, USA; e Molecular Biology Unit, Department of Basic Sciences, Faculty of Medicine, University of Kinshasa, Kinshasa, Democratic Republic of Congo; f Department of Internal Medicine, Faculty of Medicine, Our Lady of Kasayi University, Kananga, Democratic Republic of Congo; g Research Department, Association of Future African Neurosurgeons, Immeuble Nziko, Rue Marie Gocker, Yaounde, Cameroon; h University of Ghana Medical School, College of Health Sciences, University of Ghana, Accra, Ghana

**Keywords:** comorbidities, Democratic Republic of Congo, hypertensive heart disease, management

## Abstract

**Background:**

Low-and middle-income countries account for over 80% of the global burden of cardiovascular disease (CVD). The Sub-Saharan African region is the most affected by CVD. Hypertensive Heart Disease (HHD) is a common complication of hypertension which is prevalent in the Democratic Republic of Congo (DRC). Our study aimed to identify the comorbidities associated with and the management of HHD in the DRC.

**Materials and methods:**

This cross-sectional analysis was done at a 200-bed tertiary hospital in Kinshasa, DRC from January to December 2019. Data were collected retrospectively from patient records and missing values were generated by multiple imputations and the pooled values were used for data analysis. Bivariate and multiple correlation regression were used and odds ratios were generated.

**Results:**

34 (56.7%) of the 60 patients were male. The mean age was 63.2 ± 9.7 years, the mean BMI was 25.5 ± 5.0 kg/m^2^, and 90.0% of patients were unemployed. Patients had dyslipidemia (43.3%), stroke (31.7%), and diabetes (10.0%). Mean serum creatinine (1.4 ± 0.1 mg/dL), HDL (56.7 ± 18.4 mg/dL), LDL (119.3 ± 30.5 mg/dL), and median urea (24.0, IQR: 63.5 mg/dL) levels were abnormal. HDL was a predictor of high blood pressure (*P* < 0.01). 83.3% of patients took statins, 78.3% took ACE inhibitors, and 70.0% took aspirin.

**Conclusion:**

Congolese HHD patients have multiple comorbidities. Efforts should be focused on increasing access to care through early diagnosis, early referral, and low-resource appropriate management.

## Introduction

Cardiovascular diseases (CVDs) are the leading cause of morbidity and mortality worldwide, accounting for an estimated 423 million cases and 17.9 million deaths annually. These diseases contribute significantly to the global health burden, particularly in low- and middle-income countries. 80% of the 423 million CVD patients worldwide live in low- and middle-income countries (LMICs)^[1,2]^. LMICs experience worse patient outcomes and account for 80% of CVD deaths globally^[3]^.

**HIGHLIGHTS**

- This study identified a high prevalence of dyslipidemia (43.3%), stroke (31.7%), and abnormal cholesterol levels in unemployed Ghanaian adults (mean age 63 years).
- This study found that low HDL cholesterol levels were a significant predictor of high blood pressure in this population.
- Despite unemployment, a high proportion of patients adhered to medication regimens for blood pressure control (ACE inhibitors 78.3%) and cholesterol management (statins 83.3%).

In addition to the global disparities in the burden of CVDs, there is an equal disparity in the prevalence and outcomes of CVD among LMICs. Sub-Saharan Africa has the second highest prevalence rates of CVD and risk factors in the world^[2]^. Hypertension, the most common CVD risk factor in Sub-Saharan Africa, is a major determinant of poor patient outcomes^[4]^.

A common complication of hypertension is hypertensive heart disease (HHD)^[5]^. The risk factors for developing hypertensive heart disease include old age, African ethnicity, being overweight, physical inactivity, excess dietary salt intake, smoking, alcohol intake and comorbidities such as diabetes mellitus^[6]^. In Sub-Saharan Africa, the rise in CVD is driven by changing lifestyles including physical inactivity, higher consumption of alcohol and tobacco, and diets rich in saturated fats, salt, and sugar, driven by urbanization, westernization and socioeconomic development. As many as 20 million individuals have hypertension In Sub-Saharan Africa and black patients tend to develop more severe HHD^[7]^.

The burden of hypertension and HHD in Africa can be mitigated if CVD specific health systems strategies are implemented. Strategies include improving means of early detection, enhancing access to effective drug therapy, promoting treatment adherence, increasing public awareness and adopting food based dietary guidelines^[7-10]^. The treatment of patients with hypertension is cost-effective and can avert 250 000 deaths in Africa^[5,11]^.

With 84 million inhabitants, the Democratic Republic of Congo (DRC) is the fourth most populous country in Africa and 68% of its population is aged 25 years or less^[12]^. At least 2.2 million Congolese have hypertension and this number is expected to increase considerably as the population grows older^[2]^. Congolese physicians and patients face difficulties in the management of hypertension. As many as 78% of Congolese patients have uncontrolled hypertension and hypertension is commonly associated with other CVD risk factors in the general population^[13-15]^. Often, patients do not receive the adequate medical treatment because of therapeutic inertia or the patients’ inability to purchase the drugs^[15]^. Consequently, they often present to the hospital for the management of acute HHD complications^[16]^.

Previous Congolese studies have focused on the socioeconomic and clinical aspects of hypertension or heart failure in the general population^[13-16]^. To our knowledge, no study has evaluated the landscape of HHD in the DRC. Here, we perform a cross-sectional analysis of adult HHD patients in a tertiary hospital in the DRC to identify comorbidities and socioeconomic factors associated with the presence and presentation of HHD.

## Materials and methods

Data were collected retrospectively from electronic patient records at a tertiary hospital facility located in Kinshasa, the capital of the DRC. All patients aged 18 years or older and diagnosed with hypertensive heart disease at the hospital from January to December 2019 were consented and included in the study. Patients that were not diagnosed with HHD or could not confirm HHD diagnosis based on clinical examination were excluded.

Information on the patients’ sociodemographic characteristics, history, clinical examination, workup, and treatment was extracted. Radiologic examinations included electrocardiography (EKG) to assess cardiac rhythm, ventricular function and for other radiographic abnormalities, and chest x-rays to assess cardiomegaly, pulmonary edema, and other radiographic abnormalities. Physical examinations included cardiac auscultation and measurements of blood pressure. Blood tests included tests for creatinine, urea, electrolytes, lipid profile and INR. Treatment regimens, including antihypertensive medications, statins, and antiplatelet agents, were documented.

The data from this study are openly available in the Open Science Framework at DOI 10.17605/OSF.IO/5E7DU. This study is reported in line with the STROCSS 2021 Guideline Checklist.^[17]^

## Statistical remarks

We performed missing data analysis to identify incomplete variables and the pattern of missingness. The threshold for the detection of missing values was set at 0.5%. We then generated data for the missing independent variables by multiple imputations using a regression model. Minimum values were set to avoid the generation of unreasonable values by the regression model. The imputed values were averaged to generate pooled values, and all subsequent data analysis was done using the pooled values.

Data analysis was done using SPSS v26 (IBM, U.S.A.). Continuous data were expressed as means and standard deviations, while categorical data were expressed as frequencies and percentages. We equally calculated the skewness and kurtosis of the data (Supplemental File: Table 1, available at: http://links.lww.com/MS9/A708). Next, we created Q-Q plots (Supplemental File: Figure 1, available at: http://links.lww.com/MS9/A708). Then, we performed the Kolmogorov-Smirnov and Shapiro-Wilk tests to help determine if the data significantly deviates from a normal distribution (Supplemental File: Table 2, available at: http://links.lww.com/MS9/A708). Based on the results of the Q-Q plots and the variable class, we analyzed bivariate relationships with non-parametric tests. Multiple correlation regression analysis was used to analyze variables that showed statistically significant associations during bivariate analysis. We generated odds ratios with 95% confidence intervals, and a *P* value of <0.05 was statistically significant.

## Results

Patients were diagnosed with HHD from January to December 2019. 8 variables had missing values: Creatinine, HDL cholesterol, INR, LDL cholesterol, serum potassium, total cholesterol, triglycerides, and urea (Table 1).

**Table 1:** Missing value analysis for the hypertensive heart disease data

Variable	Number of patients missing data (percentage) N = 60	Mean	Standard deviation
Creatinine (mg/dL)	10 (16.7)	1.2	0.09
HDL a cholesterol (mg/dL)	17 (28.3)	56.6	19.9
INR b	48 (80)	1.4	0.1
LDL c cholesterol (mg/dL)	10 (16.7)	120.3	31.1
Serum potassium (mmol/L)	13 (21.7)	3.8	0.9
Total cholesterol (mg/dL)	14 (23.3)	176.4	42.8
Triglycerides (mg/dL)	17 (28.3)	92.8	58.3
Urea (mg/dL)	10 (16.7)	29.9	26.4

^a^ High-density lipoprotein.

^b^ International normalized ratio.

^c^ Low-density lipoprotein.

The separate variance t-test revealed statistically significant association between both weight and height with total cholesterol, triglyceridemia, LDL cholesterol, urea, and creatinine. The missing value pattern of analysis confirmed that the data were not missing completely at random (Table 1 and Table 3).

The mean age was 63.2 ± 9.7 years, and the mean body mass index (BMI) was 25.5 ± 5.0 kg/m^2^. 34 (56.7%) patients were men, 48 (80.0%) were married, 40 (66.7%) were college graduates, and 54 (90.0%) were employed. 54 (90.0%) had a history of diabetes, 26 (43.3%) had dyslipidemia, 19 (31.7%) had suffered a stroke, and no (0%) patients had a history of coronary artery disease (Table 2).

**Table 2:** Sociodemographic characteristics of Congolese patients with hypertensive heart disease

Characteristic	Female	Male	Total	Odds ratio for female/male (95% CI)	*P* value
Number	26 (43.3)	34 (56.7)	60 (100)		
Age (years)	59.7 ± 10.8	65.8 ± 8.0	63.2 ± 9.7		0.01*
Body mass index (Kg/m^2^)	24.3 ± 5.3	26.4 ± 4.6	25.5 ± 5.0		0.43
Marital status					0.05
Married	18 (30.0)	30 (50.0)	48 (80.0)		
Single	4 (6.7)	0	4 (6.7)		
Widow(er)	4 (6.7)	4 (6.7)	8 (13.3)		
College graduate	6 (10.0)	34 (56.7)	40 (66.7)	0.23 (0.11-0.47)	<0.01**
Employed	20 (33.3)	34 (56.7)	54 (90.0)	0.77 (0.62-0.95)	0.03*
History					
Coronary artery disease	0	0	0		
Diabetes	0	6 (10.0)	6 (10.0)	1.21 (1.04-1.42)	0.02*
Dyslipidemia	16 (26.7)	10 (16.7)	26 (43.3)	0.26 (0.09-0.77)	0.13
Stroke	3 (5.0)	16 (26.7)	19 (31.7)	6.82 (1.72-27.1)	0.03*
Lifestyle					
Alcohol use	13 (21.7)	14 (23.3)	27 (45.0)	0.70 (0.25-1.96)	0.50
Smoking	0	0	0		
Presenting symptoms					
Chest pain	9 (15.0)	0	9 (15.0)	0.65 (0.49-0.87)	<0.01**
Dyspnea	12 (20.0)	23 (38.3)	35 (58.3)	2.43 (0.85-7.00)	0.09
Orthopnea	0	7 (11.7)	7 (11.7)	1.26 (1.06-1.49)	0.01*
Palpitations	3 (5.0)	6 (10.0)	9 (15.0)	1.64 (0.37-7.30)	0.51

^*^ *P* < 0.05.

^**^ *P* < 0.01.

**Table 3:** Pooled mean value for the laboratory values of Congolese patients with hypertensive heart disease

Variable	Mean	Standard deviation	Change in mean (pooled data − original data)
Creatinine (mg/dL)	1.4	0.1	+0.2
HDL a cholesterol (mg/dL)	56.7	18.4	+0.1
INR b	1.4	0.1	0
LDL c cholesterol (mg/dL)	119.3	30.5	−1.0
Serum potassium (mmol/L)	3.8	0.9	0
Total cholesterol (mg/dL)	173.9	40.2	−2.5
Triglycerides (mg/dL)	98.2	54.8	+5.4
Urea (mg/dL)	35.6	38.4	+5.7

^a^ High-density lipoprotein.

^b^ International normalized ratio.

^c^ Low-density lipoprotein.

**Figure 1.**
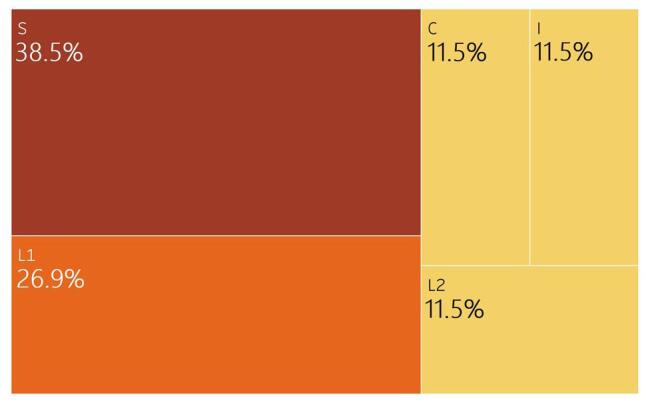
EKG abnormalities among Congolese patients with hypertensive heart disease.

On cardiac auscultation, S3 or S4 sounds were identified in three (5.0%) patients. 26 (43.3%) patients had abnormal electrocardiograms (EKGs) (Fig. 1).

S: sinus bradycardia, complete left bundle branch block, and secondary repolarization abnormalities, L1: left ventricular hypertrophy only, I: isolated ventricular extrasystole and a negative T wave, L2: left anterior hemiblock, isolated supraventricular extrasystoles, and left ventricular hypertrophy, C: complete left bundle branch block, left ventricular hypertrophy, abnormalities of R wave progression in V1-V3, and secondary repolarization disorders.

**Figure 2.**
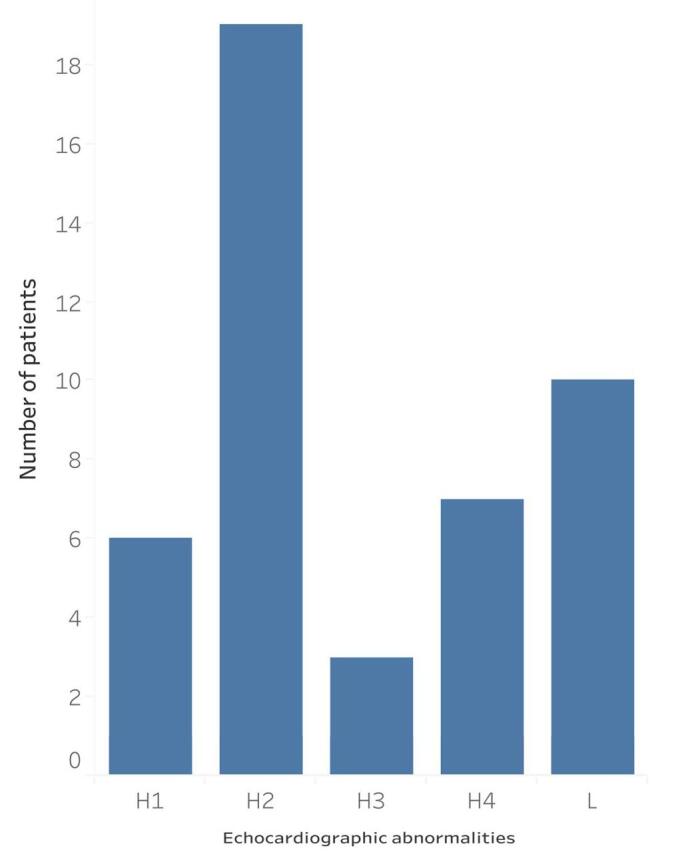
Echocardiographic abnormalities in Congolese hypertensive heart disease patients.

45 (75.0%) patients had echocardiographic abnormalities. Hypertensive and ischemic heart disease with mid-range ejection fraction (HFmrEF) was the most common finding (n = 19) followed by left ventricular hypertrophy with diastolic dysfunction (n = 10) (Fig. 2).

H1: hypertensive and ischemic heart disease with collapsed left ventricular ejection fraction, H2: hypertensive and ischemic heart disease with mid-range ejection Fraction, H3: hypertensive and ischemic heart disease with preserved left ventricular ejection fraction, H4: hypertensive heart disease with mid-range ejection fraction, L: left ventricular hypertrophy with diastolic dysfunction.

15 (25.0%) patients had radiographic abnormalities: 9 (15.0%) had cardiomegaly, 4 (6.7%) had cardiogenic pulmonary venous hypertension, and 4 (6.7%) had sequelae of lung disease.

**Figure 3.**
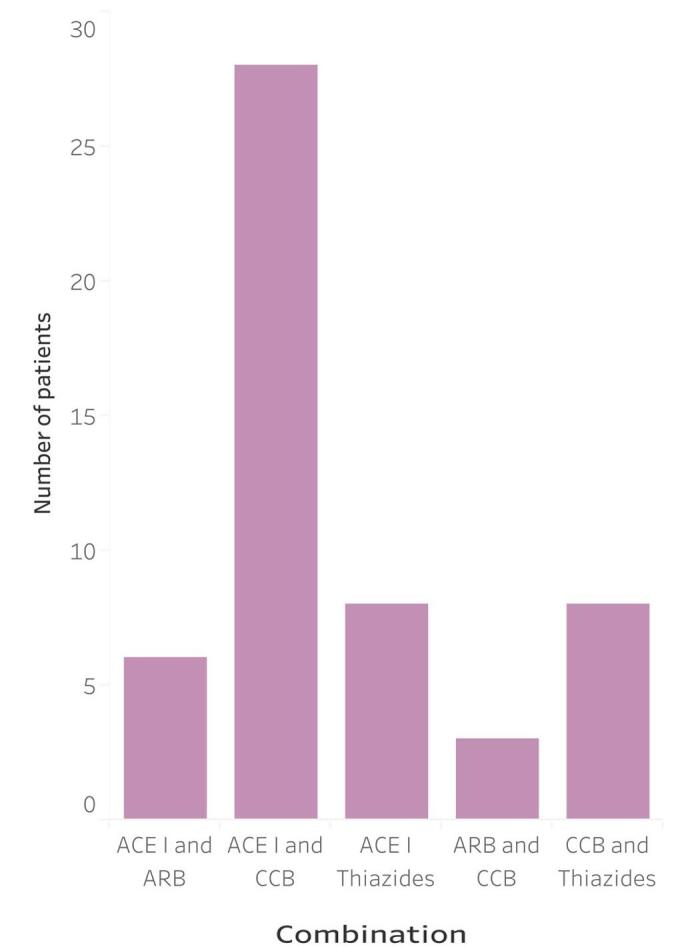
Two-drug antihypertensive drug combinations for hypertensive heart disease Congolese patients.

50 (83.3%) patients were taking statins, and 42 (70.0%) patients were taking low-dose aspirin regularly. 47 (78.3%) were on ACE inhibitors, 43 (71.7%) on beta-blockers, 35 (58.3%) on calcium channel blockers, 15 (25.0%) on angiotensin II receptor blockers, and 11 (18.3%) on thiazides. The antihypertensive bitherapy the patients were taking is illustrated in (Fig. 3).

ACE I: angiotensin converting enzyme inhibitor, ARB: angiotensin II receptor blocker, CCB: calcium channel blocker.

The mean systolic blood pressure (SBP) was 146.5 ± 15.5 mmHg and the mean diastolic blood pressure (DBP) was 84.2 ± 7.8 mmHg. The SBP was positively correlated with the BMI (*R* = 0.26, *P* = 0.049), LDL cholesterol (*R* = 0.64, *P* <0.01), and total cholesterol (*R* = 0.59, *P* < 0.01). However, SBP was negatively correlated with HDL cholesterol (*R* = −0.35, *P* = 0.02), creatinine (*R* = −0.60, *P* < 0.01), and urea (*R* = −0.65, *P* < 0.01) (Table 4).

**Table 4:** Correlation between blood pressure values and other variables

Variable	Systolic blood pressure		Diastolic blood pressure	
	Correlation	*P* value	Correlation	*P* value
Parametric^a^				
Age	0.20	0.13	0.01	0.92
Body mass index	0.26	0.049*	0.10	0.46
HDL^b^ cholesterol	−0.35	0.02*	0.82	<0.01**
Non-parametric^c^				
Creatinine	−0.60	<0.01**	−0.38	<0.01**
INR^d^	0.60	0.04	0.80	<0.01**
LDL^e^ cholesterol	0.64	<0.01**	0.27	0.05
Serum potassium	0.07	0.65	0.02	0.92
Total cholesterol	0.59	<0.01**	0.34	0.02*
Triglycerides	−0.25	0.11	0.08	0.60
Urea	−0.65	<0.01**	−0.25	0.82

^a^ Pearson’s correlation.

^b^ High-density lipoprotein.

^c^ Spearman’s rho correlation.

^d^ International normalized ratio.

^e^ Low-density lipoprotein.

* *P* < 0.05.

^**^ *P* < 0.01.

The overall correlation between BMI, creatinine, HDL cholesterol, LDL cholesterol, total cholesterolemia, and urea with SBP was *R* = 0.83 (*R*^2^ = 0.70, *F* = 111.55, *P* <0.01). HDL cholesterol (*P* <0.01), LDL cholesterol (*P* <0.01), total cholesterol (*P* <0.01), and urea (*P* <0.01) were all unique predictors of SBP. Similarly, the overall correlation of creatinine, HDL cholesterol, INR, and total cholesterol was statistically significant (*R* = 0.80, *R*^2^ = 0.64, *F* = 118.64, *P* <0.01). HDL cholesterol (*P* <0.01) and INR (*P* <0.01) were the only unique predictors of DBP.

## Discussion

Most patients were men, married, and employed. Female patients were younger and had a smaller BMI than male patients. Almost half of patients had dyslipidemia and 1-in-10 had diabetes. Stroke was the most common CVD associated with HHD, and the most frequent presenting symptom was dyspnea. Less than half of patients had an abnormal EKG and three-quarters had an abnormal echocardiography. Creatinine, HDL, INR, LDL, total cholesterol, and urea levels were all abnormal. In addition, the SBP and DBP were slightly elevated. Blood pressure control reduces the risk of HHD progression when the SBP is <120 mmHg^[18]^. Control of blood pressure and HHD progression can be achieved with antihypertensive drugs but equally by the implementation of interventions aimed at vulnerable populations such as patients with uncontrolled cholesterol, diabetics, elderly patients, and patients with a recent history of CVD complication^[19]^.

Creatinine, HDL cholesterol, and total cholesterol were individually correlated with SBP and DBP in our study. In the multivariate models, HDL cholesterol was the only predictor of SBP and DBP with a statistically significant correlation. HDL is inversely correlated with left ventricular hypertrophy (LVH) in both men and women, and should be kept within the normal range to reduce the risk of progression of HHD^[19]^. Additionally, the HDL-to-total cholesterol ratio is a widely available and cost-effective predictive tool for the progression of CHD^[20]^. In contrast, the direct measurement of LDL is complex and less standardized^[21]^. The concomitant management of blood pressure and cholesterol levels generally requires a two-drug antihypertensive drug combination together with a lipid-lowering agent^[21]^. 83.3% of Congolese patients in this sample were on statins and more than 88.3% were taking a combination of ACE inhibitors, calcium channel blockers, or diuretics. The most prescribed antihypertensive bitherapy were ACE inhibitors with calcium channel blockers, followed by ACE inhibitors with thiazides and calcium channel blockers with thiazides.

HFmrEF and left ventricular hypertrophy with diastolic dysfunction were the most common echocardiographic findings. HFmrEF which is characterized by a moderately reduced ejection fraction (i.e., 40-49%) reflects a compromise in the left ventricular systolic function due to the combined effects of hypertension and ischemic heart disease. This finding suggests that management strategies should focus on optimizing blood pressure control, addressing ischemic burden, and using heart failure therapies that improve outcomes in patients with mid-range ejection fraction. Monitoring for progression to heart failure with reduced ejection fraction (HFrEF) is essential. Regarding left ventricular hypertrophy with diastolic dysfunction, the study findings support the need for aggressive blood pressure control, lifestyle modifications, and medications that improve diastolic function and reduce left ventricular hypertrophy. The predominance of hypertensive heart disease in various stages highlights the significant burden of hypertension in this patient population. Early identification is crucial to prevent disease progression and complications. Of note, echocardiography picked up more HHD cases than EKG. EKG is the preferred screening tool for HHD. It is inexpensive and has a high specificity but a low sensitivity^[22,23]^. Diagnostic concordance between EKG and echocardiography is dependent on the EKG criteria used. Previous studies have found that EKG-echocardiography diagnostic concordance is higher when using the Cornell product instead of the Sokolow-Lyon^[24,25]^. The discordance between EKG and echocardiography can equally be due to advanced age, female gender, and the use of antihypertensive medication^[26]^. Although echocardiography is more likely to ascertain LVH than an EKG, doing so does not change the management of HHD patients. For this reason, the indications of echocardiography are limited to symptomatic congestive heart failure patients, children or teenagers, and patients with chronic and uncontrolled hypertension^[27]^. 46.7% patients had signs of hypertensive and ischemic heart disease on the echocardiogram and 41.7% had a collapsed or midrange left ventricular ejection fraction. In the neighboring Republic of Congo, 49.4% of HHD patients have LVH on echocardiographic examination with the left ventricular ejection fraction ranging between 58-90%^[28]^.

Previous studies have illustrated the difficulties faced in the detection and management of HHD in the DRC and neighboring countries. Lulebo *et al* found that primary health care facilities conducted little on-the-job training of nurses and other health workers on the management of CVD, and only one in four nurses was aware of clinical guidelines and the necessary patient education for HHD^[29]^. At all healthcare levels in the DRC, a majority of patients with hypertension have uncontrolled hypertension, in large part due to a lack of awareness, lack of timely diagnosis, and financial barriers to afford treatment^[30]^. In the Republic of Congo, HHD patients commonly presented at a late stage when heart failure sets in, often due to poor adherence to treatment and financial constraints^[31]^. In rural Zambia, a high prevalence of hypertension and HHD was observed, further confirming that LMICs gradually shift from a predominance of communicable diseases to increasing burdens of non-communicable diseases, even in remote areas^[32]^. This epidemiological transition requires attention and appropriate clinical and patient education at all levels of the health system. Leveraging community health workers and digital health technologies (e.g., mobile health) may improve diagnosis and continuity of care of HHD^[33,34]^. Further, health systems barriers such as medical and non-medical costs, geographic distance, and awareness promotion and education ought to be addressed in a systematic manner to improve access to care for patients in the DRC and other LMICs^[35]^.

This study has some limitations. First, the design of this study does not permit us to determine causal relationships between dependent and independent variables. Second, this being a cross-sectional study, we could not establish temporal relationships between the variables. However, we chose this study design because it was the most feasible given our resources, which is a common struggle in conducting studies in LMICs by LMIC researchers. Third, the small sample and the potential effect of confounders increases the risk of type II error in our study. However, we reduced the effect of confounders with multiple correlation regression analysis. Finally, the models could have been improved with appropriate data transformations as follows: positive skewness – high kurtosis data like total triglycerides, urea, and creatinine were log transformed; weight and LDL were square root transformed; potassium and INR were Box-Cox transformed; height and DBP were reflected before being log transformed. Despite these limitations, our study gives an insight into the landscape of HHD in the DRC.

Congolese HHD patients are obese and have high blood pressure values despite taking antihypertensive drugs. HDL cholesterol can be used as a good predictor of hypertensive systolic and diastolic blood pressures in the DRC. More should be done to diagnose and manage CVD risk factors in the Congolese population. This study may inform the management of patients with HHD in the DRC, and future studies and interventions should focus on increasing access to care for HHD patients.

## Data Availability

All datasets generated during and/or analyzed during the current study are publicly available and available upon reasonable request.

## Research registration unique identifying number (UIN)

clinicaltrials.gov (NCT06524440).

## Study location

Monkole Mother and Infant Hospital Center which is a 200-bed tertiary hospital facility located in Kinshasa, the capital of the DRC.
